# On the networked architecture of genotype spaces and its critical effects on molecular evolution

**DOI:** 10.1098/rsob.180069

**Published:** 2018-07-04

**Authors:** Jacobo Aguirre, Pablo Catalán, José A. Cuesta, Susanna Manrubia

**Affiliations:** 1 Grupo Interdisciplinar de Sistemas Complejos (GISC), Madrid, Spain; 2 Programa de Biología de Sistemas, Centro Nacional de Biotecnología (CSIC), Madrid, Spain; 3 Departamento de Matemáticas, Universidad Carlos III de Madrid, Leganés, Madrid, Spain; 4 Instituto de Biocomputación y Física de Sistemas Complejos (BIFI), Universidad de Zaragoza, Zaragoza, Spain; 5 UC3M-BS Institute of Financial Big Data (IFiBiD), Universidad Carlos III de Madrid, Getafe, Madrid, Spain

**Keywords:** genotype–phenotype map, network-of-networks, adaptive multiscapes, molecular promiscuity, phenotypic plasticity, punctuated dynamics

## Abstract

Evolutionary dynamics is often viewed as a subtle process of change accumulation that causes a divergence among organisms and their genomes. However, this interpretation is an inheritance of a gradualistic view that has been challenged at the macroevolutionary, ecological and molecular level. Actually, when the complex architecture of genotype spaces is taken into account, the evolutionary dynamics of molecular populations becomes intrinsically non-uniform, sharing deep qualitative and quantitative similarities with slowly driven physical systems: nonlinear responses analogous to critical transitions, sudden state changes or hysteresis, among others. Furthermore, the phenotypic plasticity inherent to genotypes transforms classical fitness landscapes into multiscapes where adaptation in response to an environmental change may be very fast. The quantitative nature of adaptive molecular processes is deeply dependent on a network-of-networks multilayered structure of the map from genotype to function that we begin to unveil.

## 1. Introduction

Gradualism posits that any profound change in nature is the result of minor cumulative modifications due to the action of slow but sustained processes. First proposed in the framework of Geology at the end of the eighteenth century by James Hutton, gradualism underlies Charles Lyell's theory of uniformitarianism [1], which formed one of the conceptual pillars of Charles Darwin's evolutionary theory soon after [2]. Ever since, gradualism has been a powerful concept in the qualitative interpretation of evolutionary change.

The gradualistic view of evolution has been challenged at the macro- (fossil record), meso- (ecological) and micro- (molecular) scales. In the 1970s, analyses of data in the fossil record revealed an unanticipated pattern of evolutionary stasis in the morphological change of species that was punctuated by sudden jumps, leading to the theory of punctuated equilibria [3]. The mechanistic models proposed to generate that dynamical pattern are not unique, though the endogenous organization of the biosphere may have played a main role [4,5]. At present, punctuated equilibrium is understood as an alternation of periods with insignificant change (stasis) punctuated by rapid speciation, which may, however, extend over a few hundred thousand years and result from complex evolutionary dynamics [6]. Analogies between macroevolution and evolutionary ecology were suggested on the basis that the degree of complexity observed in the spatial and temporal organization of both systems might be reflecting a network-like organization close to critical points [7], the latter resulting from a combination of external drivers and internal adaptive responses. Research in this century has unveiled a large number of cases where smooth environmental changes may indeed trigger sudden and irreversible ecological responses [8,9]. The complex interaction between natural systems and varying environments remains an open question of critical relevance. The factors that make ecosystems respond smoothly or drastically to a weakly evolving environment have attracted special interest, as there are direct implications in the relationship between humans and a changing biosphere that could eventually reach a hazardous tipping point [8,10,11].

**Figure 1.**
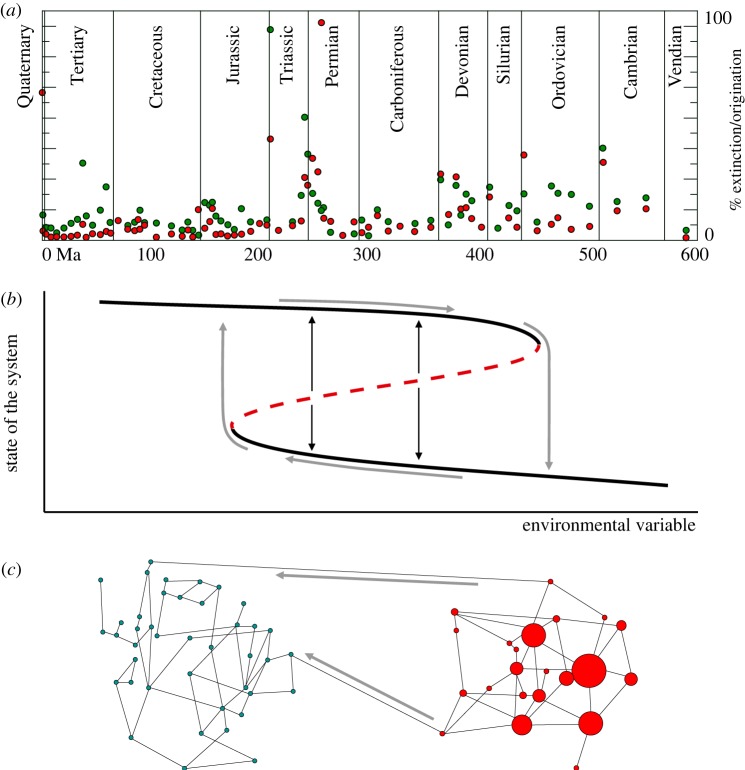
Punctuated behaviour in macroevolution, ecology and molecular dynamics. (*a*) Non-uniform pattern of extinctions (red symbols) and originations (green symbols) in the last 610 Myr (0 is present). Each point corresponds to a geological epoch, vertical lines separate geological periods, as indicated. The vertical axis gives the percentage of extinction/origination per estimated diversity at each epoch and per million years. Data from [12], geological epochs and periods as in [13]. (*b*) Minor changes in environmental variables might cause large, nonlinear responses in the state of a variety of systems. In some cases, two stable solutions (black curves) coexist with an unstable solution (red curve) for a range of values of a control parameter. The trajectories of systems might follow the path indicated by the grey arrows as that parameter increases, suffering a sudden jump from the upper to the lower branch. Hysteretic behaviour appears and prevents the recovery of the initial state when the environmental variable is reverted. When the system is initiated close to the unstable branch, it may attain any of the two possible stable solutions (black thin arrows). (*c*) In the genotype space, nodes represent genotypes and links correspond to single mutational moves. Heterogeneous molecular populations contain a set of genotypes with variable abundances, the latter represented through circle size. Fitter regions in genotype space might be difficult to find if there are few mutational incoming pathways (grey arrows). The population might be trapped in the red phenotype for a relatively long time (stasis) when compared with the transition to the new state once suitable mutations have appeared (punctuation).

The formal description of non-uniform dynamics in natural systems is advancing concomitantly with the number of examples supporting and clarifying the theoretical framework (figure 1). Shifts in ecosystems have been formally described as bifurcations leading to hysteretic behaviour and also as critical transitions. Analogous to fluctuations close to critical points, the so-called early warning signals can anticipate such catastrophic responses [14]. Empirical evidence of this phenomenon with a single species has been described in laboratory populations of yeast [15], while there is a variety of well-documented examples in ecology, such as the hysteretic loss and recovery of charophyte vegetation at lake Veluwe [16], the desertification of the Sahara [9,17], the loss of transparency in shallow lakes [18] or the dynamics of woodlands in Tanzania [19]. A thorough description of this phenomenology is a hard task, as it involves a wide variety of time scales and biological levels—many of them organized as complex networks—that interact in a complex manner [20]. At the molecular level, the architecture of the genotype–phenotype map entails non-uniform evolutionary dynamics [21]. In particular, it has been shown that the steady accumulation of point mutations under a selective pressure acting on the phenotype yields population dynamics characterized by stasis (when sequences explore neutral regions) punctuated by phenotypic changes (when a fitter phenotype is found) [22]. Smooth changes at the level of sequences do not preclude sudden adaptive changes at the level of function: well-motivated models support that, like the state of ecosystems, changes in genomic composition might be sudden, irreversible and unavoidable [23]. These dynamics have been also documented in the *in vivo* evolution of a virus, influenza A, which shows a seasonal pattern where expansion of genotypic diversity predates the finding and fixation of strains with novel antigenic properties that escape immune detection [24,25].

Despite mounting evidence, the long tradition of relating small changes in sequences to gradual changes in organisms and populations persists, often in a tacit way. A significant example is Wright's adaptive landscape [26], which appears as a direct consequence of gradualistic thought and counts among the most powerful metaphors in Biology, one that has conditioned evolutionary thinking for almost a century [27]. Indeed, the image of a relatively smooth landscape, where populations adapt by going uphill, are trapped in mountain peaks and remain isolated from other possibly higher fitness maxima by deep valleys, often appears as the way in which adaptation proceeds. This picture implies a smooth and continuous genotype-to-phenotype (GP) map and a space of low dimensionality. Thanks to advances in our knowledge of the molecular structure of populations, we now know of important elements missing in most theoretical adaptive landscapes. For example, genotypes of similar fitness are found to form extensive networks that occasionally traverse the genotype space, especially in spaces of high dimensionality [28]. The GP map actually entails a many-to-many correspondence: genotypes are plastic and may yield different phenotypes when expressed in different environments. This latter case seems to be much more common than previously thought, meaning that the co-option of promiscuous, secondary gene functions [29] is likely a common adaptive mechanism. From a formal viewpoint, therefore, the complexity of the GP map implies that fitness landscapes should be visualized as high-dimensional and interwoven sets of networks that unfold into multiple layers under environmental change [30]. New techniques, in particular the use of deep sequencing and powerful massive ways to evaluate the fitness of individual genotypes, represent a breakthrough in the empirical characterization of the complex genotype-to-phenotype-to-function relationship [31,32]. Interestingly, the network-of-networks structure of genotype spaces described in realistic, though artificial, models is also emerging in empirical characterizations of the diversity of molecular populations [33].

Adaptive evolutionary systems, such as large-scale evolution, ecology or (molecular) populations, share deep analogies that can be likely ascribed to their networked architecture plus a non-trivial relationship between exogenous drivers and endogenous responses. In this review, we will focus on molecular dynamics, which is the least studied of those three profoundly entangled levels of description of the evolutionary process. The architecture of genotype spaces and the dynamics of evolving molecular populations are two sides of the same coin. The heterogeneous structure of genotype spaces and its apparently hierarchical organization as a multilayered network of networks explains, among others, punctuated dynamics [22], drift and switch transitions [24], genomic shifts [23] or Waddington's genetic assimilation [30,34].

## 2. Genotype networks

Kimura [35,36] introduced the concept of neutral evolution in order to explain why many mutations observed in RNA, DNA or proteins do not affect fitness. Neutrality implies that the GP map is not one-to-one, but many-to-one, consistently explaining the high level of polymorphism observed in natural populations. Soon after Kimura's seminal work, navigability was hypothesized as an essential requirement to guarantee the evolvability of molecular populations [37]. Usually, navigability is believed to rely on the existence of sufficiently large *neutral networks* (NNs) of genotypes [38] since these should permit the neutral drift of populations and a sustained exploration of alternative phenotypes without a detrimental decrease in fitness. An NN is formed by all genotypes that map into the same phenotype. As fitness is linked to phenotype, all genotypes in an NN are implicitly assumed to have the same fitness. Genotypes are the nodes of such networks, and links correspond to single mutational moves. In its simplest and most popular definition, a mutational move stands for a point mutation. Neutral networks can have one or several connected components. Navigability on NNs has been subsequently identified as a robust property of computational models [22,39–41] and natural molecular populations [25,42–44].

The actual set of genotypes visited by an evolving population, however, is rarely neutral. Nearly neutral mutations are common in finite populations [45], augmenting their adaptive ability. In fact, any finite mutation rate entails that populations are heterogeneous in sequence, phenotype and function, such that the potential set of genotypes of a population includes genotypes of different fitness, which constitute the actual navigable network. In certain cases, as for ensembles of fast mutating replicators such as quasi-species [46,47], the maintenance of a large phenotypic diversity and the permanent exploration of the genome space become critical survival strategies [48]. We will call *genotype network* the network of visited genotypes and, by extension, any potentially navigable network in the space of genomes, regardless of the fitness or phenotype of its nodes.

### 2.1. Neutral networks in computational genotype–phenotype maps

**Figure 2.**
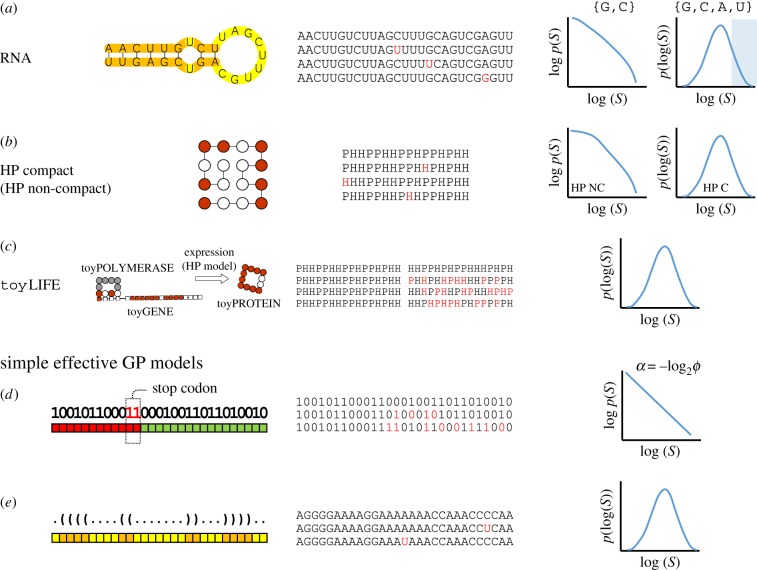
Some examples of simple GP maps. For each model, and from left to right, we depict an example phenotype, some of the sequences in its neutral network (mutations that do not change the phenotype are highlighted in red), and the schematic functional form of the probability distribution *p*(*S*) of phenotypes sizes *S* found in computational or analytical studies. (*a*) RNA sequence-to-minimum-free-energy secondary structure. Mutations that do not disrupt the secondary structure appear with different probability in loops or stacks. In two-letter alphabets, the distribution of phenotype sizes is compatible with a power-law function [49], while in four-letter alphabets *p*(*S*) is well fit by a lognormal distribution [50]. For long sequences, only the right-most part of *p*(*S*) can be seen under random sampling of the genotype space [50] (shaded). (*b*) The HP model, in its compact (as in the figure) or non-compact versions, has been studied as a model for protein folding. In non-compact versions, the distribution *p*(*S*) has a maximum at *S* = 1 and decays with a fat tail [51], while in compact versions *p*(*S*) resembles a lognormal distribution [52]. (*c*) toyLIFE is a minimal model with several levels. HP-like sequences are read and translated to proteins that interact through analogous rules to break metabolites. The *p*(*S*) of toyLIFE is compatible with a lognormal distribution [53]. (*d*,*e*) Effective models where phenotype is defined in relation to the composition of sequences allow to analytically calculate the functional form of *p*(*S*). Two examples are (*d*) Fibonacci's model [54], where *p*(*S*) follows a power-law distribution and (*e*) an RNA-inspired model [55] which yields a lognormal distribution of *p*(*S*).

Neutral networks have been quantitatively characterized in a number of computational GP maps (figure 2). RNA sequences fold into a minimum free energy secondary structure that we can take as a proxy for its phenotype [38,56]. Given a sequence length, the number of minimum free energy secondary structures is much smaller than the number of sequences, leading to large NNs [38,50,57–61]. In models of protein structure, such as the HP model [62], proteins are formed by strings of two amino acids: hydrophobic (H) and polar (P). As in RNA, this sequence will fold into a minimum free energy structure, and there are many more sequences than structures [51,63–65]. In a completely different model, gene regulatory networks possess an evolvable architecture [66] that gives rise to several temporal gene expression patterns, which represent the phenotype. Again, many interaction topologies representing the genotype give rise to a much smaller number of gene expression patterns [28,67]. Neutral networks also appear in metabolic processes. If we consider the genotype as a list of enzymatic reactions and the phenotype as the set of metabolic sources on which an organism can survive, it is found that many genotypes can actually survive in a set of environments [41,68–70]. Finally, NNs have also been observed in complex models that include cellular population dynamics and several levels from genotype to phenotype [71], in more abstract GP maps, such as the polyomino model of polymer self-assembly [72,73], toyLIFE—a multilevel model of a simplified cellular biology [53,74]—and in simplified combinatorial models [54,55].

Most NNs studied in the literature share a remarkable number of structural properties [28,75]:

1. Most phenotypes are rare, and only a few of them are very common. Specifically, the probability of finding a phenotype when sampling uniformly at random among all of them follows a lognormal distribution for a wide variety of models [50,55,74] and a power law for some special cases [49,54,55]. Therefore, a small fraction of the largest phenotypes contains most genotypes, such that in practice those are the only ones visible to natural selection [50,59,76]; together with the asymmetry in the mutual accessibility of two phenotypes [58,77], that property causes a form of (entropic) trapping in genotype space [74,76,78,79].
2. The degree of a node in an NN, defined as the number of one-mutant neighbours that belong to the same NN (aka its genotypic robustness), is a heterogeneous quantity, although its distribution is often unimodal [28,39,61]. Additionally, the average degree of an NN is proportional to the logarithm of the size of the network [55,60,61,80].
3. These NNs are assortative, at least for phenotypes defined through minimum-energy principles [61,81,82]. In an assortative network, genotypes are connected to other genotypes of similar degree, and this correlation in genotypic robustness causes canalization [83], leads to phenotypic entrapment [79] and enhances evolvability [80].
4. Neutral networks of common phenotypes percolate genotype space. In other words, we can find two genotypes expressing the same phenotype with a sequence similarity comparable to that of two randomly chosen genotypes [28,84].
5. Most large phenotypes are one mutation away from each other, such that genotypes yielding every common phenotype can be found at the boundary of any large NN [28,53,65,84]. As a result, the search for new phenotypes among common ones is a fast process.

The space of genotypes can be depicted in this context by a number of interconnecting NNs when each node is projected in a horizontal (quasi-) neutral layer whose vertical position represents its fitness value. In this multilayer perspective [85,86], intralayer connections between individual nodes represent neutral mutations, while interlayer connections represent mutations that beneficially (upwards) or deleteriously (downwards) affect fitness [87]. It is, however, important to keep in mind that this representation is suitable only if the GP map is approximated as a many-to-one relationship, since it fails to include the frequent correspondence between one genotype and several possible (environment dependent) phenotypes, as will be discussed in §§5 and 6.

### 2.2. Genotype networks in genotype-to-function maps

The GP map is at best a toy representation of the relationship between genotype and function, though it hopefully captures some of its statistical properties. Computational studies suggest that structural properties of GP maps are largely independent of the precise definition of phenotype [88,89] and of details of specific models [28,75], and data to assess whether GP maps are a sufficiently accurate representation of genotype-to-function maps—which represent a qualitative step forward—is mounting. Advances in experimental techniques have allowed to study the structure of the genotype-to-fitness mapping through either experimental evolution studies [33,90–93] or high-throughput data [32,44,94]. The resulting experimental fitness landscapes confirm and extend the picture of molecular evolution gained through the computational study of simple GP maps, showing the presence of many quasi-neutral (eventually navigable) regions [95] and decaying correlations between phenotypes as the mutational distance increases [96]. Natural fitness landscapes have an intermediate degree of ruggedness, they are neither smooth nor random, therefore revealing an important role of epistasis in shaping the topological properties of genotype networks and in defining eventually accessible genomic pathways for molecular adaptation [44,90,97,98].

Fitness landscapes have been theoretically explored through models where phenotypes need not be explicitly defined and, instead, a fitness value is associated with each genotype. This representation is closer to data retrieved through empirical evolutionary experiments. The NK model [99] has proved to be especially useful to generate an underlying landscape with realistic degrees of ruggedness [24,92]. Furthermore, it is relatively simple, only depending on two parameters—the length of the sequence *N* and the level of ruggedness *K*—but versatile enough to model fitness landscapes with natural properties such as epistasis, multiple fitness peaks and local optima [100].

It turns out that topological differences between genotype networks, obtained through data that map genotype to function, and NNs, as described in the previous subsection, are only cosmetic. It can be shown that spaces of genotypes endowed with the structure of the NK model are also organized as a network of networks, that is, as a set of genotype networks qualitatively equivalent to NNs connected through a limited number of pathways [101]. The structural properties of genotype networks, visualized as a multilayered network of networks, define a particular class of dynamics for populations evolving on such architecture.

The following sections are devoted to the not yet fully understood interaction between the topology of genotype networks and the evolutionary dynamics of heterogeneous populations—at least from the formal viewpoint of dynamical systems. We begin by synthesizing current evidence to demonstrate that three different dynamical situations (competitive transitions between different regions of an NN [102], punctuated molecular adaptation [22] and genomic shifts under varying environments [23]) can be described within a unique conceptual and theoretical framework. In subsequent sections, we will show how the latter framework can be extended to include the many-to-many inherent structure of GP maps and environmental changes.

## 3. Population dynamics on neutral networks

In order to describe mathematically the evolution of heterogeneous populations on NNs, let us recall that many dynamical processes occurring on a network can be expressed as3.1
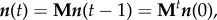
where 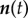 is a vector whose components are the population of individuals at each node at time *t* and **M** is an evolution matrix that contains the particulars of the dynamical process (see box 1).

Box 1. **Dynamics of replicators on a fitness landscape.**

The evolution of a population of asexually replicating individuals on a fitness landscape described as a genotype network can be written as3.2
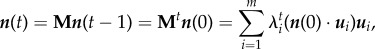
where 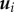 and λ_*i*_ are the eigenvectors and eigenvalues of the evolution matrix **M** and *m* is the number of nodes of the genotype network; 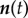 has length *m*. We order the eigenvalues and eigenvectors such that λ_*i*_ ≥ λ_*i*+1_. If **M** is primitive, Perron–Frobenius theorem for non-negative matrices ensures that, over time, the system evolves towards an asymptotic state characterized by the (unique) first eigenvector 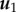. More precisely3.3
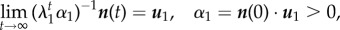
regardless of the initial condition 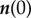. The components of 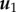 (all of them guaranteed to be strictly positive by the same theorem) are proportional to the fractions of the total population at each node once the process has reached mutation-selection equilibrium, while its associated eigenvalue λ_1_ represents the asymptotic growth rate of the population. The transient dynamics towards equilibrium is ruled by the subsequent eigenvalues, but in most cases the time to reach the equilibrium state verifies *t*_eq_ ∝ [ln(λ_1_/λ_2_)]^−1^, since the contributions of higher-order terms are suppressed exponentially fast [103].

In a population of replicators that mutate with probability 0 < *μ* < 1 per genotype and replication cycle, matrix **M** can be decomposed as^1^
3.4
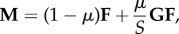
where **F** is the diagonal matrix *F*_*ij*_ = *f*_*i*_*δ*_*ij*_, *f*_*i*_ being the fitness (i.e. replication rate) of node *i*; **G** is the adjacency matrix of a connected graph, whose elements are *G*_*ij*_ = 1 if nodes *i* and *j* are connected and *G*_*ij*_ = 0 otherwise; and *S* stands for the maximum number of neighbours of a genotype [23]. When replicators are sequences of length *l* whose elements are taken from an alphabet of *A* letters, the size of the genotype space is *m* = *A*^*l*^ and *S* = *l*(*A* − 1).

Matrices such as **M** in (3.4) are guaranteed to be primitive if the network **G** is connected and the diagonal of **F** is strictly positive.

Dynamics on a single NN is a particular case for which the fitness components are *f*_*i*_ = *f* if *i* is a genotype in the NN and 0 otherwise—all sequences replicate at a rate *f*.

For the sake of illustration, let us start by considering a simple fitness landscape with a single viable phenotype. The genotypes yielding the latter constitute an NN and all remaining genotypes have zero fitness. Consider genotypes as sequences of length *l* whose elements are taken from an alphabet of *A* letters. Nodes represent different sequences and links connect those sequences differing only in one letter. The evolution of a population through the space of genotypes due to mutations is here limited to the NN—or to its largest connected component in case the NN is disconnected. An evolution matrix that models such a dynamical process is3.5
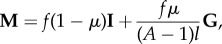
where **I** is the identity matrix and **G** is the adjacency matrix of the connected network, with elements *G*_*ij*_ = 1 if nodes *i* and *j* are connected and *G*_*ij*_ = 0 otherwise. The genotypic robustness of a node is proportional to its degree *k*_*i*_, defined as the number of genotypes one-mutation away that are on the network, 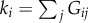. **M** describes a population that every time step replicates at each node at a rate *f* > 1, each daughter sequence leaving the node with probability 0 < *μ* < 1 and surviving with probability *k*_*i*_*μ*/(*A* − 1)*l* [103], with *k*_*i*_ the degree of the parental node. If we define *k*_min_, *k*_max_ and 〈*k*〉 as the smallest, largest and average degree of that NN, respectively, we obtain *k*_min_ < 〈*k*〉 ≤ *γ*_1_ < *k*_max_ for any heterogeneous network, *γ*_1_ being the largest eigenvalue of the adjacency matrix **G** [104]. In the case of two-letter alphabets, *A* = 2, *γ*_1_ is bounded from above by the logarithm of the number of genotypes in an NN [105]. *γ*_1_ also equals the average degree of the population at equilibrium, *κ*, so the former inequality implies *κ* > 〈*k*〉, indicating that the population selects regions with connectivity above average on the NN. This fact shows a natural evolution towards mutational robustness, because the most connected nodes are those with the lowest probability of experiencing lethal mutations. Nonetheless, the population might get trapped in regions of lower connectivity if *Nμ* < 1 [106]. The tendency towards robustness does not preclude evolutionary innovation, though. On the contrary, NNs relevant in evolution span large regions in genome space [50], with the result that they can be more robust and at the same time more evolvable [80,107,108]. A positive correlation between neutrality and evolvability stems from the fact that NNs are very interwoven: for example, all common RNA structures of length *l* can be found within a small radius of a randomly chosen sequence in genotype space —a property known as ‘shape space covering’ [84,109]. The mutual proximity of NNs in genome space (the so-called NN apposition [58,110]) has been observed empirically. Two remarkable examples are ribozymes and viruses. Indeed, two RNA sequences with independent origins can fold and function as different ribozymes when their sequences are forced to evolve to increase their similarity, eventually differing in only two nucleotides [42]; diffusion on NNs is instrumental to permit innovation and immune escape in influenza A [24].

The eigenvectors of the adjacency matrix **G** are also eigenvectors of the evolution matrix **M**, as can be seen in equation (3.5). Their respective eigenvalues, *γ*_*i*_ and λ_*i*_, are different—albeit related through λ_*i*_ = *f*(1 − *μ*) + *γ*_*i*_*fμ*/(*A* − 1)*l*. As a consequence, in NNs the asymptotic state of the system only depends on the topology of the NN, and parameters such as the mutation rate *μ* or the sequence length *l* exclusively affect the transient dynamics towards equilibrium [103,106]. This result cannot be extrapolated to more general fitness landscapes, where both the equilibrium state of the population and the transient dynamics depend in a non-trivial fashion on network topology and genotype fitness [103] (cf. equations (3.2) and (3.4) in box 1).

Heterogeneity in the degree of the nodes, or equivalently in genotypic robustness, and the assortativity inherent to many NNs have important consequences in the dynamics of populations. Soon after the hypothesis of the molecular clock [111] was put forward, variations in genotypic robustness were suggested as an explanation for its unexpected overdispersion [112]. If networks are furthermore assortative, the probability that the population leaves the network diminishes the longer the time spent on it, leading to a progressive (phenotypic) entrapment. Beyond a systematic increase in the overdispersion of the process with time, assortativity entails an acceleration in the fixation rate of neutral mutations [79], invalidating the Poissonian assumption underlying the molecular clock.

## 4. Punctuated dynamics in molecular adaptation

As soon as more realistic architectures of the genotype space are considered, dynamics becomes punctuated. This fact has been highlighted in formal studies stating that GP maps based on RNA sequence-to-structure relationship naturally imply punctuation, irreversibility and modularity in phenotype evolution [21], and has been nicely illustrated in computational works [22,58,110].

The formal scenario that we use here starts at the level of genotypes, but also takes into account the non-trivial topology induced by the mapping onto phenotypes. By means of techniques that exploit the networked and modular structure of genotype spaces, we will show that the dynamical behaviour is qualitatively similar in three different situations, that is if (i) an NN has two or more regions of high connectivity linked through few possible mutational pathways, (ii) a population encounters a phenotype of fitness higher than the extant one or (iii) mutation-selection equilibrium is perturbed through an environmental change that entails a modification of the fitness landscape. Underneath the punctuated dynamics observed in those situations there is a common mechanism: a (formal) competition between regions with a high internal connectivity that are sparsely connected to one another. These highly internally connected regions may be different clusters of genotypes in a single NN, different phenotypes each characterized by its own NN, or different regions in a fitness landscape. Actually, this synthesis emerges as a generalization of processes occurring on a wide variety of biological, technological and social dynamics on networks of networks (i.e. networks connected through a limited number of connector links). This class of processes admits a description in terms of competitive scenarios where each network is defined as an independent agent struggling with the rest for a particular kind of resource [113–115]: *eigenvector centrality* (see box 2).

Box 2. **Competition for centrality in a network of networks.**

In complex network theory, the eigenvector centrality *x*_*k*_ of a node *k* in a network is defined as the *k*th component of the eigenvector of its adjacency matrix **G** corresponding to the largest eigenvalue *γ*_1_ [116]. The eigenvector centrality has become one of the most widespread metrics for node importance because of its wide range of applications, which include Google Pagerank [117], estimations of the professional impact of scientists [118] and journals [119], the importance of individuals in a social group [120] or of regions in the brain [121], and dynamical processes such as disease or rumour spreading (see [116] for an overview).

This measure can be generalized to other dynamical processes if **G** is replaced by another (non-negative) matrix **M**: the new eigenvector centrality is defined through 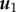, the eigenvector corresponding to λ_1_, the largest eigenvalue of **M** (see e.g. box 1). In evolutionary dynamics, the eigenvector centrality is thus the fraction of population with each genotype at mutation-selection equilibrium [103]. We use this generalization in the following.

In a network of networks, the centrality of each network is the sum of the centralities of all its nodes, normalized in such a way that the sum of the centralities of all networks is equal to one. Therefore, combining game theory and network science, we can approach the spread of the total centrality on the different networks as a zero-sum game, where players are not nodes but networks and *compete* for centrality, which is understood as a limited resource. The winnings of each competing network *α* are calculated as the total centrality *C*_*α*_ accumulated by all its nodes

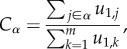
where *j* runs on the nodes of network *α* and 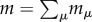 is the total number of nodes in the network of networks. The outcome of such confrontations for centrality and the time needed by the winner to prevail drastically depend on (i) the internal structure of the competing networks 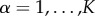, as characterized by their maximum eigenvalue λ_1,*α*_, in a way that networks with larger λ_1,*α*_ in general obtain more centrality than their competitors and (ii) the connector nodes, that is, the boundary nodes that connect one of these networks with the rest of them through connector links.

When connector links occur only through nodes with little centrality (aka peripheral connections), almost all centrality remains in the network with the largest eigenvalue λ_1,*α*_. If for some reason (e.g. an environmental change) the eigenvalue of a different network overcomes λ_1,*α*_, a sharp centrality redistribution takes place. The time to reach the equilibrium significantly increases close to that transition.

### 4.1. Metastable states and punctuation in a network-of-networks architecture

In §3, we have focused on the dynamics of populations evolving on a single NN characterized by a well-defined region of maximum connectivity. Under those conditions, the evolutionary dynamics of a sufficiently large population is smoothly canalized towards the maximally connected region of the NN [83,103,106]—something that has measurable effects on the fixation rate of neutral mutations [79]. However, there is no *a priori* reason to assume that generic NNs do not present a complex structure formed by more than one cluster of nodes with high internal connectivity and sparse connections to one another. For instance, the topology of NNs associated with RNA secondary structures recently revealed an intricate network-of-networks organization, where the different communities can be further divided into subcommunities attending to their sequence composition [122, Fig. 6]. Some of these networked communities turned out to be two mutational steps away—such mutations playing the role of what we have named connector links—and therefore required an intermediate group of genotypes for a population to move from one community to another. In this type of complex structure, the evolutionary dynamics of populations on NNs can display an alternance of metastable states (which might appear as true equilibria at short times) with periods where neutral mutations are rapidly fixed [102].

The formalism that describes competition between networks for centrality, while originally introduced in the framework of complex network theory, was recently proven to be fully applicable to the study of populations evolving in the space of genotypes [101]. The population distribution at mutation-selection equilibrium is given by the first eigenvector 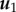 of the matrix **M** that characterizes the dynamical process, and therefore the centrality that each network competes for coincides with the fraction of organisms that populate its corresponding sequences in the asymptotic state. In general, the most populated network in the equilibrium is the one with the largest eigenvalue λ_1_ of matrix **M** (box 2).

**Figure 3.**
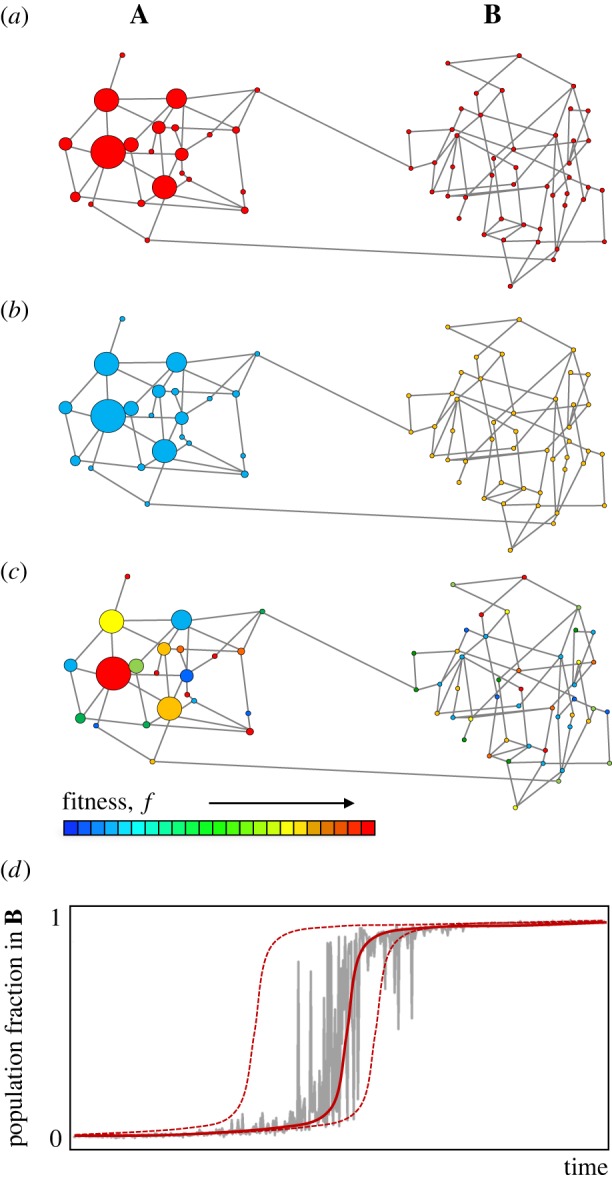
Genomic shifts result from the network-of-networks structure of the space of genotypes. Without loss of generality, we assume that λ_1,*A*_ < λ_1,*B*_ and the whole population is initially in network **A**. In (*a*–*c*), colours indicate the fitness of each node, as shown by the colour scale, and circle size is indicative of the number of individuals at each node. Though nodes in network **B** are represented with small circles, we assume they have no population initially. (*a*) Two weakly coupled regions of a unique NN. Differences in their eigenvalues only depend on differences in their topology. (*b*) Two different NNs with different fitness. The effect of fitness and topology can be separated, both affect their eigenvalues. (*c*) Two weakly connected regions in a fitness landscape. The effects of fitness and topology cannot be decoupled. (*d*) In all cases, the time of transitions is a stochastic variable, but the transition is fast once the mutational pathway is found (red curves, corresponding to different realizations of the process). In changing or noisy environments, the fitness value of each sequence might vary in time, so transitions are decorated by fluctuations (grey curve) whose strength grows as the tipping point is approached.

Let us illustrate in the simplest case how a population moves from a subnetwork with a lower eigenvalue λ_1,*A*_ to a subnetwork with a larger eigenvalue λ_1,*B*_ in the framework of competition for centrality. Figure 3*a* represents two regions of an NN weakly connected. As previously described, we have λ_1,*A*_ = *f*(1 − *μ*) + *γ*_*A*_*fμ*/(*A* − 1)*l*, and similarly for network **B**. Note that the latter network will eventually be attracting the population if the eigenvalue corresponding to its evolution matrix λ_1,*B*_ is larger than that of **A** and, as a consequence, the same applies to the adjacency matrices (i.e. *γ*_*B*_ > *γ*_*A*_). This result shows that the separating barrier only depends on the topological structure (size and connectivity) of each subnetwork. The transition to a region with higher connectivity occurs upon stochastic appearance of mutations along connecting pathways. This process is highly contingent, so the time of the punctuation is difficult to predict (red lines in figure 3*d*). Actually, too small populations might be indefinitely trapped in regions as **A** [22].

### 4.2. Drift and switch dynamics in adaptive transients

Early evidences of punctuation in molecular adaptation came from computational simulations of populations of RNA sequences evolving towards a target secondary structure [22]. Typically, populations remain on the current phenotype until a higher-fitness solution is found, that is, until one of the genotypes in the population acquires a mutation that produces a new, fitter phenotype. This event is preceded by a ‘search’ in the original phenotype during which the population accumulates neutral mutations and increases its genotypic diversity. The switch transition is not deterministic, since different phenotypes can be reached first depending on the stochastic occurrence of mutations. Once the new phenotype has been found, the transition occurs exponentially fast but, concomitantly, the population experiences a severe bottleneck that reduces its genotypic diversity. In this scenario, a new phenotype can be accessed through any genotype in the neighbourhood of genotypes of the original phenotype, though peripheral genotypes (those with a higher number of links pointing to different phenotypes, i.e. of low robustness) are more likely to act as connectors than highly robust, central genotypes [79]. This drift and switch dynamics is characteristic of any realistic GP map with a structure such as that described in §2. In the dynamical framework of competition between networks, each phenotype represents now a distinguishable network characterized by its size, connectivity and fitness level. Connector links correspond to regions of apposition between the two networks, which exist in most cases (in particular when the two phenotypes considered are common) but are difficult to find if populations are finite due to the vastness of genotype spaces and NN [30]. Also, the connector links might join regions with similar fitness but different internal connectivity, or regions with different fitness, among many other possibilities. Different paths to adaptive improvement are taken with different probability. For example, narrow neutral paths are crossed much faster than fitness valleys [123].

Figure 3*b* illustrates the situation of two phenotypes with different fitness values (i.e. replicative ability of its nodes) coupled through narrow paths. The transition to phenotype **B** might occur if λ_*B*,1_ > λ_*A*,1_ which implies that4.1
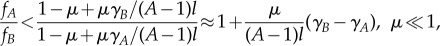
where the specific effect of fitness *f*_*i*_ and topology *γ*_*i*_ is quantified.

The survival-of-the-flattest effect represents one particular case of such competition where the two competing regions have different levels of fitness, different mutation rates (a situation that can be easily included in the framework above), and different levels of robustness [124,125], which effectively accounts for different topologies [126]. Some computational models that consider relevant features of molecular populations have been developed, leading to an improved understanding of this interesting phenomenon. The effective implementation of networks with different degrees of neutrality permits to capture competitions between RNA populations subject to selection for different folds (characterized by different neutral networks), showing how the relative advantage of either population changes sign as a function of the mutation rate [127]. Also, in a model of quasi-species characterized by bit strings, it has been shown that a discontinuous phase transition separates the regions of selection for replication and selection for robustness [128]. At odds with results emphasizing selection for robustness along evolution, models with RNA quasi-species show that high mutation rates might be evolutionarily advantageous in situations where a single RNA sequence might code for a molecular ecosystem [129]. It has been also argued that fitness landscapes with fitter and flatter regions might be behind the enigmatic richness of microbial metabolisms [130]. Epochal evolution (i.e. metastable states punctuated by rapid transitions to fitter states) have also been observed in evolutionary search algorithms, as referred to a class of optimization techniques [131,132].

The theory can be easily extended to any number of phenotypes in competition and yields a clear prediction regarding the phenotype that will be eventually attracting the population. The largest eigenvalue of any matrix **M**, λ_1_, is a fundamental quantity that synthesizes information on the topology of the underlying network, on the fitness of its nodes and on the mutation rate. These three elements combine in a non-trivial way to determine the competitive ability of a population on a given network. In this respect, a population can asymptotically displace a competitor for a number of different reasons, namely because (i) it spreads on a larger NN, (ii) its average fitness is higher, (iii) it spreads on a network with higher connectivity, (iv) it mutates at an advantageous rate with respect to its competitors or (v) any suitable combination of the previous reasons.

### 4.3. Smooth environmental changes and genomic shifts

There is empirical evidence that environmental changes affect the evolutionary dynamics of populations and their eventual fate [133]. Recalling that fitness is an environment-dependent quantity, environmental changes can be formally cast as modifications of the fitness associated with genotypes. When a genotype space is mapped to a realistic fitness landscape, smooth environmental changes can be represented as gradual modifications of the fitness value of each genotype. As phenotype is here a hidden variable, at this point we do not need to consider possible changes in phenotypic expression due to environmental variation. This possibility will be discussed later though.

Even if environmental variations are smooth, populations may eventually suffer sudden transitions in their genomic composition [23]. In the case of finite populations, there is a non-zero probability of extinction if the pathway linking the (decreasingly fit) current state of the population to a new region populated by fitter phenotypes is not found sufficiently fast [101]. The abundance and breadth of connecting pathways depends on the roughness of the landscape and on the fraction of lethal mutations, which can be put in correspondence with important variables such as the degree of heterogeneity of the corresponding genotype networks and the holeyness of the landscape [134]. These quantities tune the number of connector links between different regions with significant fitness and the centrality of their connector nodes. As a consequence of the above, fitness landscapes can be described as a network of networks formally analogous to the examples discussed previously (figure 3*c*).

Early warning signals that forecast the proximity of tipping points (and therefore of a putative extinction threshold) can be defined in analogy to studies of sudden shifts in ecology [14]. Close to those state transitions populations show flickering and hysteresis, i.e. a dependence on its previous states that causes trapping and metastability, and is eventually responsible for extinction [101].

Summarizing, facing evolutionary systems from the viewpoint of competing networks turns the space of genotypes into a network of networks at several different levels. The full consequences of this architecture are still to be understood, though they are certainly far from trivial: relevant phenomena such as robustness [135,136], synchronization [137,138], cooperation [115,139,140] or epidemic spreading [141–143] exhibit different features when their dynamics occur on a single network or on a network of networks.

## 5. The many-to-many nature of the genotype–phenotype map

Our discussion so far has assumed that each genotype corresponds to a unique phenotype. Adaptation to a new environment or selection pressure, therefore, has to be achieved through mutations, and we have discussed some of the non-trivial phenomena that appear when heterogeneous populations evolve in a complex genotype space. However, there are many cases in which genotypes express more than one phenotype, opening up new possibilities for adaptation: in any realistic realization, the GP map is many-to-many, since genotypes are able to express different phenotypes in a variety of situations. In this section, we present several examples of this phenomenon and discuss how it alters the dynamics discussed in previous sections. The reader should know that the level of formal description achieved is poorer than for dynamics on networks and has received much less attention up to now. Our feeling is that, as shown in previous sections, theory should help towards unifying processes and concepts that are treated at present as different phenomena. However, the following sections rely much more on the description of the latter than on quantitative results. A full mathematical formalism that describes at once the multilayered, network-of-networks structure of the genotype-to-function map is an open and on-going problem of the highest relevance.

### 5.1. Molecular promiscuity

Enzymes were classically thought to be highly specific: one enzyme–one substrate–one reaction. However, recent experimental data have shown that, in fact, many enzymes are able to catalyse more than one reaction, a phenomenon that has been termed catalytic or functional promiscuity [144–149]. This means one amino acid sequence corresponds to more than one phenotype. Promiscuous enzymes are not hard to find in sequence space. For example, single-site mutants of bacterial enolases can actually perform secondary functions not found in the wild-type, while maintaining their original activity [150]. Moreover, these promiscuous functions are easily evolvable: enzymes can accumulate mutations that do not alter their main function, but which radically change their secondary ones [151–153], and the activity of secondary functions can be increased several orders of magnitude with very few mutations [144,154,155].

Promiscuous activities can help enzymes evolve towards new functions. A polymorphic population of enzymes can diversify with respect to its secondary functions if they bear no fitness costs to the organism, leading to the accumulation of what has been termed cryptic genetic variation [156]. When selection pressure for a new function appears, those enzymes in the population that carry out that function as a promiscuous activity will be already functional and, in a sense, pre-adapted for it. The new function can then be improved through over-expression [147] or gene duplication that liberates one copy of the enzyme to specialize in the new function [144,148,157]. These promiscuous activities also have an effect on metabolism, connecting different metabolic pathways [148,158], and therefore enabling their gradual evolution: promiscuous enzymes can develop their secondary functions, so that certain steps in a pathway become more efficient, in turn liberating other enzymes to focus on other parts of the pathway. The evolution of metabolic pathways, therefore, can be achieved in a more parsimonious way. When a new pathway is needed, cells with promiscuous enzymes may perform the needed reactions, and give these sequences an adaptive advantage.

Functional promiscuity is not restricted to enzymes: transcription factors have been shown to bind many different motifs with comparable binding energies [32,44,148]. Also, proteins can be mistranslated [159], a process that is several orders of magnitude more common than genetic mutations, and thus at a given moment in time, some proteins will have a different amino acid sequence, with potentially different functions that can accelerate adaptation to a new function [160–162]. Some protein sequences will be more likely to yield new functions under these phenotypic mutations.

Promiscuity is also not restricted to proteins. Early computational work on RNA secondary structures [38] already suggested that RNA molecules could fold into more than one structure, and recent experimental studies have found evidence of RNA molecules that can perform more than one different function [163,164]. The best examples are ribozymes (RNA enzymes) that are able to catalyse two different reactions [42,165,166]. Computational [83,167] and experimental studies [166] suggest that secondary functions in RNA molecules can evolve as easily as in proteins, and that this functional promiscuity can spread through populations as cryptic genetic variation, accelerating the rate at which new functions are found in evolution. Even if these functions are performed marginally at first, they will give the sequence an advantage if they are selected for, and freedom to improve the new function in genotype space. In fact, theoretical models predict that promiscuous functions can help accelerate evolution towards a new function, through what has been called the look-ahead effect [160]. Although this phenomenon was originally proposed for phenotypic mutations, it is also valid for promiscuous enzymes and RNA molecules.

### 5.2. Phenotypic heterogeneity and bet-hedging

The fact that one sequence can perform more than one function is not restricted to the molecular level. At the regulatory level, for instance, expression noise is very common [168–170], due to the stochastic nature of transcription and translation and the small number of molecules involved in these processes. Expression noise leads to phenotypic heterogeneity [171,172], where two genetically identical genotypes can, under the same conditions, express two different phenotypes at the cellular level. Although expression noise is inherent to the biochemical process of building the phenotype from the genotype, cells can control it to some level [169,173–175], and they can also use it to their advantage [171,172,176]. For instance, genotypes can evolve a stochastic switching mechanism that enables them to alternate between two different phenotypes, a phenomenon that has been termed bet-hedging [177]. At a given moment in time, a fraction of the population will express one phenotype and the rest another one. Each phenotype is typically advantageous in one environment and disadvantageous in another, and so the ability to switch between them is adaptive under some conditions [178]. Typical examples of bet-hedging are bacterial competence [179] and persistence [180]. Bet-hedging is a common mechanism that can also emerge in evolution experiments [181]. These strategies would not be possible without functional promiscuity.

### 5.3. Phenotypic plasticity

Another piece of this puzzle comes from phenotypic plasticity, a well-known phenomenon in which a genotype is able to express different phenotypes in different environments [182]. Notice the difference from phenotypic heterogeneity as discussed above: phenotypic plasticity is only unveiled when an environmental cue appears. In fact, strategies such as bet-hedging arise when the cost of developing a plastic response—which is able to sense the environment—is so high that it becomes disadvantageous [178].

Phenotypic plasticity has been known for a long time in multicellular organisms, but it appears at the unicellular and molecular level as well. Proteins are not only promiscuous: they can also carry out different functions in different environments, a phenomenon that is called moonlighting [183,184]. One classical example is crystallin lenses, enzymatic proteins whose function becomes structural when expressed at very high concentrations [185]. The same gene can also express different proteins through alternative splicing [184]. RNA molecules can fold into different structures at different temperatures, performing different functions [186]. RNA thermometers, as they are called, can be designed computationally [187]. Gene regulatory networks have different spatio-temporal expression patterns when exposed to different environmental inputs [188–190], and metabolic systems are able to survive on different food sources [68–70].

A plastic population will be able to automatically survive in a new environment, if it expresses a viable phenotype. Once in the new environment, it might spread through the new fitness landscape, maybe losing its original plasticity. Many theoretical and computational studies of plasticity and its relationship with adaptation have been proposed [191–197], although most of them do not include the complexities of the GP map that we have discussed in our previous sections. They assume that phenotypes that are close in trait value to the ones present in the population will always be achievable through mutations. Therefore, the discussion of when and how phenotypic plasticity will be promoted cannot account for the biases induced by more or less abundant phenotypes, asymmetric connections between them and other factors discussed so far in this review, which could affect how easily plasticity is developed. There are, however, some computational studies that explicitly model GP maps, focusing on RNA molecules [83] and gene regulatory networks [189,198].

## 6. Hints for a dynamical theory of many-to-many genotype–phenotype maps

### 6.1. Promiscuity redefines the fitness landscape

How do we integrate all of these data into the framework we have been discussing so far in this review? The presence of phenotypic noise or functional promiscuity (at the molecular or regulatory level) implies that a single genotype, in a given environment, will express more than one phenotype in a probabilistic manner. Therefore, the effective fitness of the genotype will be an intermediate value related to the fitness associated with each phenotype. Naively, one could guess that the fitness *f*_*i*_ of sequence *i* would be 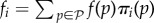, where 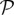 is the set of all phenotypes, *f*(*p*) is the fitness of phenotype *p* and *π*_*i*_(*p*) is the probability that sequence *i* expresses phenotype *p* (alternatively, *π*_*i*_(*p*) represents the fraction of the homogeneous population with genotype *i* expressing phenotype *p*). To illustrate one such case, consider a population of RNA sequences that perform their function by interacting with a ligand. Under the minimum free energy mapping usually considered in the literature, all RNA sequences expressing the optimal structure as their minimum free energy are assigned the same fitness. Including promiscuity, however, alters this fitness function. Two sequences belonging to the same NN have different compositions, and this variation leads, in general, to differences in their folding energies and also in the repertoire of structures with which they are compatible [199]. Differences in the folding energy entail differences in the average time spent in the minimum free energy secondary structure for each specific sequence. In this situation, a more accurate definition of fitness takes it as proportional to the time spent in the optimal secondary structure. Therefore, two sequences belonging to the same NN have different fitness values under this more realistic quantification of their function.

However, a careful investigation of the underlying (stochastic) population dynamics reveals that the simple average above is not of general applicability, as the next example illustrates. Consider a homogeneous population of cells expressing a certain phenotype with probability *p*, and another one with probability 1 − *p*. The replication rate *β* of both phenotypes is the same, but the second phenotype has a higher death rate *δ*_2_ > *δ*_1_—i.e. it has a lower fitness, defined as the difference between birth and death rates, *f* = *β* − *δ*. There is no mutation in this example. Whenever any cell replicates, the daughter cell expresses one of the two phenotypes with the aforementioned probabilities, regardless of the mother's phenotype. Calling *m*_1_(*t*) and *m*_2_(*t*) the number of cells of each type at time *t*, we can use results from birth–death processes theory to derive the following system of ordinary differential equations:6.1
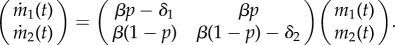
We diagonalize the system to obtain its largest eigenvalue (and thus, the asymptotic fitness of the population):6.2
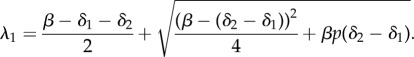
With some algebra, we can show that λ_1_ > (*β* − *δ*_1_)*p* + (*β* − *δ*_2_)(1 − *p*), the latter being the result of the naive guess above, i.e. that the average fitness of the population is the weighted average of the fitness of the visited phenotypes, where weights are the probability that a genotype expresses each phenotype. The discrepancy arises, in this case, because cells expressing the second phenotype die more often. As a result, the population has an overrepresentation of cells expressing the more stable phenotype: their fraction in the population is actually greater than *p*.

Despite the differences between the two examples discussed, it appears that the effect of promiscuity can be accounted for by properly redefining the fitness landscape. Each example, however, will need to be carefully examined to correctly translate its dynamical details to a suitable definition of fitness.

### 6.2. Dynamics of plastic phenotypes under frequent environmental changes

Phenotypic plasticity means that the same genotype expresses different phenotypes in different environments, such that different evolution matrices have to be considered in each of the environments (see box 3). This is equivalent to considering one GP map per environment, and switching between them when the environment changes. To fix ideas, suppose we have two different environments alternating every generation, with associated matrices **M**_1_ and **M**_2_. Then the evolution of the population will be given by the largest eigenvalue of the matrix **M**_2_**M**_1_ and the asymptotic state of the population turns out to be an orbit with period 2, as long as some conditions are fulfilled. Both matrices (and their product) must be primitive (see box 1). This happens, for instance, if all nodes have positive fitness or if, after removal of the zero-fitness nodes, none of the two networks breaks down into different connected components. If this condition is not met, the asymptotic state will depend on the initial condition. Likewise, even if all nodes have positive fitness but the fitness of some of them is very small, the population can get trapped in metastable states for very long times. But one can also imagine that alternating environments can have the opposite effect, namely, that the transit of certain pathways strongly hindered in both environments when kept constant may be facilitated by their alternation.

Box 3. **Dynamics of replicators on a shifting fitness landscape.**

The framework introduced in box 1 can be extended to account for environmental changes. For the sake of simplicity, we will just consider the case in which the environment alternates between two states, but generalizations of this are self-evident. The fitness of every node needs not be the same in each environment, and as a result the evolution matrices of both environments (we will denote them by **M**_1_ and **M**_2_) will be different.

Let us begin by exploring the case in which, starting in environment 1, we alternate environments every generation. Then the equation for the evolution of the population reads6.3
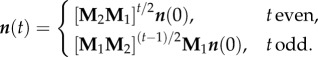
This means that, in general, the evolution of the population will be dominated by the largest eigenvalue of the matrix **M**_2_**M**_1_ at even times and of the matrix **M**_1_**M**_2_ at odd times, regardless of 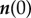. (Starting from environment 2 would only swap the parity of times, but not the general results.)

Interestingly, the eigenvalues of cyclic permutations of a product of matrices are the same, and the corresponding eigenvectors are easily related to each other. Thus, if λ_1_ is the largest eigenvalue of **M**_2_**M**_1_ and 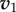 its corresponding eigenvector, then the eigenvector of matrix **M**_1_**M**_2_ will be 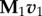, so the asymptotic population will grow as λ^*t*/2^_1_ and the fraction of population will cycle through6.4
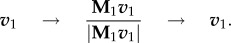

The case in which environments change following a random pattern is particularly interesting. In this case,6.5
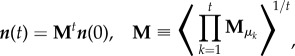
where *μ*_*k*_ ∈ {1, 2} is a discrete random process whose dynamics is prescribed (for instance, it can take each of the two values with a certain probability, or *μ*_1_ can take any value with a certain probability and swap every time step with another probability). The expected value is to be taken over realizations of this process. The largest eigenvalue of **M** and its corresponding eigenvector will determine the asymptotic behaviour of the population. Mathematically, this process is not fully characterized yet, but it is not difficult to carry out its numerical implementation.

This analysis can be extended to more complicated alternating patterns of the two environments, the only difference being that the asymptotic state will exhibit a longer period. For instance, if environments change according to the pattern 112112112 … , and λ_1_ and 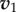 are the largest eigenvalue and its corresponding eigenvector of the matrix **M**_2_**M**^2^_1_, then the population will grow as λ^*t*/3^_1_ and the fraction of population will cycle through
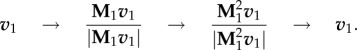


**Figure 4.**
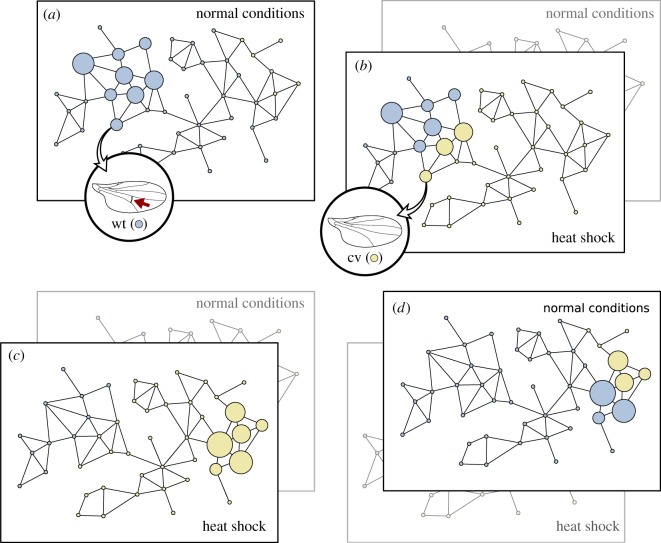
Waddington's genetic assimilation under the light of genotype networks. Each layer of the network represents a different environment. Here, there are two environments: normal conditions and heat shock. As in previous figures, circle size is proportional to the number of individuals populating that node—small circles represent unpopulated nodes. The colour of each node represents now its phenotype, instead of its fitness. Note that every genotype appears in both layers, and that connections between them are the same in both environments: the only property that changes is the phenotype. (*a*) A population of flies develops wings with a cross-vein (the wild-type phenotype, wt, blue) when bred in normal conditions. (*b*) When exposed to heat shock during development, some of the flies in the original population develop new wings without cross-veins (the cross-veinless phenotype, cv, yellow). (*c*) Breeding the flies under heat shock and then selecting for those flies expressing the cross-veinless phenotype, the population drifts towards a new part of genotype space, exploring a new neutral network (or possibly increasing fitness in the new environment). (*d*) After some time, the population is bred again in normal conditions, and some flies in the population keep expressing the cross-veinless phenotype. Their phenotype has been genetically assimilated.

A qualitative representation of this idea was already proposed in the form of adaptive multiscapes [30] (figure 4). It was shown there that the evolutionary phenomena introduced by phenotypic plasticity, such as Waddington's genetic assimilation [34], could be easily understood in terms of a multilayered network of genotype networks. Genetic assimilation is a very interesting phenomenon. In Waddington's experiment, a plastic population of flies was exposed to a new environment, in which they expressed a different phenotype (called cross-veinless). They were selected for this new phenotype under the new environment, so they spread through the genotype network in the way we have discussed in §4. After some time, when the population was brought back to the original environment, some of the individuals kept the cross-veinless phenotype, instead of reverting to the wild-type (figure 4). The phenotype that originally appeared only plastically was now being expressed without environmental changes: it had become genetically assimilated. Adaptive multiscapes help in the qualitative understanding of the molecular mechanisms underlying genetic assimilation, among others, since the population dynamics sketched in box 3 suffice to explain it.

## 7. Discussion and prospects

A large body of current evidence shows that the gradualistic view of evolution is at odds with the mechanisms operating at the molecular level, where discontinuous changes and fast pre-adaptations are the rule rather than the exception. We have presented three basic mechanisms with a strong effect on the evolutionary dynamics of biomolecules: fast exploration of new phenotypes by heterogeneous populations spread over neutral networks, competition between different networks for population (the evolutionary counterpart of eigenvalue centrality) and plasticity of phenotypes. But ubiquitous and general as they may be, these are by no means the only ones. Several other mechanisms and phenomena have been left out from our framework.

The first one has to do with mutations. The most parsimonious change in a genome is represented by point mutations. All through this review, we have shown how even these minor changes frequently cause major phenotypic modifications. The evolution of genomes, however, is often driven by mutational mechanisms that substantially modify them, such as gene duplication or horizontal gene transfer (HGT). The latter will potentially cause effects of magnitude larger than point mutations, and therefore entail still stronger effects on phenotypes and functions. The structure of genomes, especially the existence of universal regularities in the distribution of genomic elements [200], speaks about dominant mechanisms beyond organismal adaptation [201,202]. Gene sharing through HGT has played a main role in the adaptation of microorganisms [203] and is so common in microbial evolution that it has led to the idea of network genomics [204]. The reconstruction of gene-sharing networks for viruses [205] has uncovered a hierarchical and modular structure that drastically changes our view of viral species as well-defined entities. Instead, the topology of such networks reveals an utmost plastic system where genes behave as highly mobile pieces, and where not only adaptation but also evolutionary innovations might be strongly promoted through combinatorial processes—especially in viruses with segmented genomes [206]. This plastic view of the genome can be straight forwardly extended to cellular organisms.

Secondly, we have not included any kind of sexual reproduction nor recombination—of which HGT is a particular case. Though recombination might slow-down evolution under strong selection [207], in most of its forms it is a powerful enhancer of the search for novelty [208]. This power is very well illustrated in experiments of DNA shuffling [209], where a chimaeric cephalosporin created from recombination of four different ones achieves a 270-fold increase of resistance to antibiotic—compared to the eightfold increase achieved by the best cephalosporin created through point mutations alone. On top of that, the interplay between recombination and the genotype–phenotype map may induce a fascinating disruptive dynamics that resembles sympatric speciation [210], so speciation—one of evolution's major themes—may not be properly understood unless recombination is suitably incorporated in our dynamical models. However, this cannot be done if size- and frequency-dependent evolution operators are not introduced, because the probability that a recombination event takes place depends on the relative presence in the population of the sequences to be recombined. The lack of a suitable framework to describe this complication leaves any ‘ecological’ interaction between molecules or genes out of the picture. This is probably the weakest point of the network formalism—one that is of paramount importance to tackle in future work.

Even if we constrain ourselves to the range of applications to which the formalism we are advocating does apply, its actual implementation is not free from serious difficulties. To begin with, the vastness of genotype spaces makes it impossible to explore any realistic genotype–phenotype map in depth. This is a handicap that will not be solved with more powerful computers, so we need to turn to an alternative description of evolutionary dynamics. Fortunately, all models of the genotype–phenotype map share a set of common properties regardless of the details. This situation is similar to the one faced by Statistical Physics in its aim to go from microscopic models to macroscopic description, and so it can be dealt with in a similar vein. If details do not matter, we may try to build a mesoscopic description in which phenotypes, rather than genotypes, are the basic elements of our dynamical framework, and in which microscopic details are subsumed in an effective, possibly non-Markovian stochastic dynamics [79].

We also need to figure out how to incorporate promiscuity and environment in our evolutionary picture, in a way that does not require the running of specific simulations for each particular case. If a mesoscopic description is to be made, any change in the environment would entail a full reconfiguration of the network of phenotypes, thus affecting not only the phenotype that the population currently occupies but also the transitions between different phenotypes—hence the evolutionary pathways. A way to incorporate the effect of the environment would be through a multilayer formalism for networks [85,86], where different layers would correspond to different environments. Generalizing the dynamics described here to a multilayer network is as yet an open problem.

## Endnote

1 In [23,101] a slightly different form of this equation was used, with the matrix product (**F G**) instead of (**G F**) in the second term on the right. While the former can be interpreted as a filtering criteria for the stability of mutants (fitness is applied once mutation has occurred), the latter represents more precisely the evolutionary process here described (fitness affects replication rates and mutation occurs concomitantly). The qualitative results of both expressions are equivalent, in practice only differing in the specific moment when the population state is measured.

## Data accessibility

This article has no additional data.
